# Clinical features and management of a severe paradoxical reaction associated with combined treatment of Buruli ulcer and HIV co-infection

**DOI:** 10.1186/1471-2334-14-423

**Published:** 2014-07-30

**Authors:** Franck Wanda, Patrick Nkemenang, Genevieve Ehounou, Marie Tchaton, Eric Comte, Laurence Toutous Trellu, Isabelle Masouyé, Vanessa Christinet, Daniel P O’Brien

**Affiliations:** Médecins Sans Frontières, Akonolinga, Cameroon; Medical Unit, Médecins Sans Frontières, Geneva, Switzerland; Department of Dermatology, University Hospitals of Geneva, Geneva, Switzerland; Manson Unit, Médecins Sans Frontières, London, UK; Department of Infectious Diseases, Geelong Hospital, Geelong, Australia; Department of Medicine and Infectious Diseases, Royal Melbourne Hospital, University of Melbourne, Melbourne, Australia

**Keywords:** Mycobacterium ulcerans, Buruli ulcer, HIV, Paradoxical reaction, Antibiotics, Antiretroviral treatment, Prednisolone

## Abstract

**Background:**

In West and Central Africa Buruli ulcer (BU) and HIV co-infection is increasingly recognised and management of these two diseases combined is an emerging challenge for which there is little published information. In this case we present a severe paradoxical reaction occurring after commencing antibiotic treatment for BU combined with antiretroviral therapy for HIV, and describe its clinical features and management. This includes to our knowledge the first reported use of prednisolone in Africa to manage a severe paradoxical reaction related to BU treatment.

**Case presentation:**

A 30 year old immunosuppressed HIV positive man from Cameroon developed a severe paradoxical reaction 24 days after commencing antibiotic treatment for BU and 14 days after commencing antiretroviral therapy for HIV. Oral prednisolone was successfully used to settle the reaction and prevent further tissue loss. The antiretroviral regimen was continued unchanged and the BU antibiotic treatment not prolonged beyond the recommended duration of 8 weeks. A second small local paradoxical lesion developed 8 months after starting antibiotics and settled with conservative treatment only. Complete healing of lesions occurred and there was no disease recurrence 12 months after commencement of treatment.

**Conclusions:**

Clinicians should be aware that severe paradoxical reactions can occur during the treatment of BU/HIV co-infected patients. Prednisolone was effectively and safely used to settle the reaction and minimize the secondary tissue damage.

**Electronic supplementary material:**

The online version of this article (doi:10.1186/1471-2334-14-423) contains supplementary material, which is available to authorized users.

## Background

*Mycobacterium ulcerans* causes severe destructive lesions of skin, soft-tissue and bone mediated through its potent exotoxin mycolactone that are known as Buruli ulcers (BU). They are most commonly found in Central and West Africa but have been reported from Australia, Asia, South America and the Pacific [1]. It predominantly affects children in rural and remote regions and often results in permanent disability. Co-infection with HIV is increasingly recognised and is thought to result in more severe disease and slower healing times following treatment [2–4]. Paradoxical reactions have recently been recognised to complicate up to 20% of patients receiving antibiotics for BU, [5] and can lead to significant secondary tissue destruction [6]. Prednisolone has been used to successfully minimize tissue destruction from severe paradoxical reactions in Australia, [7] but its use in Africa for this indication has not been reported. It is not known whether the incidence of paradoxical reactions is affected by BU/HIV co-infection or antiretroviral treatment (ART), and there is a lack of information to guide their management in BU/HIV co-infected patients [8].

In this case we present a severe paradoxical reaction occurring in a patient co-infected with BU/HIV after commencing antibiotic treatment for BU concomitantly with ART for HIV. We describe its clinical features and management including to our knowledge the first reported use of prednisolone to manage a severe paradoxical reaction related to BU treatment in Africa.

## Case presentation

### Case details

A 30 year old man living in rural Cameroon presented to the Buruli ulcer treatment center in Akonolinga, Cameroon, in January 2013 with a 7-month history of plaque-like lesions over the lateral aspect of this left ankle and posterior aspect of his left leg both of which later ulcerated. The ulcer on the lateral aspect of the left ankle measured 14 cm x 9 cm with some small areas of necrosis and inflammation in the surrounding tissue. The second ulcerative lesion on the left leg measured 6 cm x 4 cm with minimal surrounding inflammation. Xrays of the left ankle and left leg revealed no evidence of osteomyelitis deep to the lesions. His weight was 65 kg and Body Mass Index 25. Further examination was unremarkable with specifically no clinical evidence of co-infection with tuberculosis and a chest X-ray was normal.

He had been previously diagnosed elsewhere with HIV infection and received 2 months of ART from January 2012 but had then interrupted it. A HIV antibody test on presentation to Akonolinga was positive and a CD4 count was 212 cells/mm^3^. Baseline viral load was not available.

The diagnosis of BU was made on the basis of a positive AFB stain and compatible histopathology showing a granulomatous infiltrate on tissue biopsies, and a positive IS2404 PCR and AFB stain on swab specimens, of both the ankle and leg lesions.

He was hospitalised and commenced on BU treatment with oral rifampicin 600 mg daily and intramuscular streptomycin (STM) 1 gram daily on 9/1/13. Within 24 hours he developed a fever to 39.5 degrees and a generalised pustular and itchy rash. A diagnosis of a STM hypersensitivity reaction was made and the STM was replaced by clarithromycin 500 mg twice daily. Prednisolone 30 mg orally daily was also given for 10 days and the reaction resolved.

**Figure 1:**
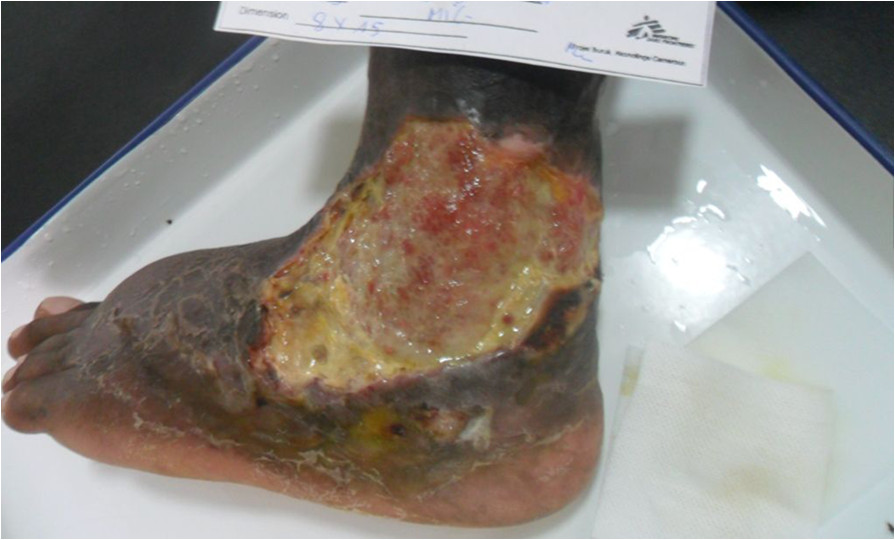
**BU ulcer on lateral aspect of the left ankle complicated by a severe paradoxical reaction with extending necrosis and swelling of skin and subcutaneous tissue of the forefoot.**

**Figure 2:**
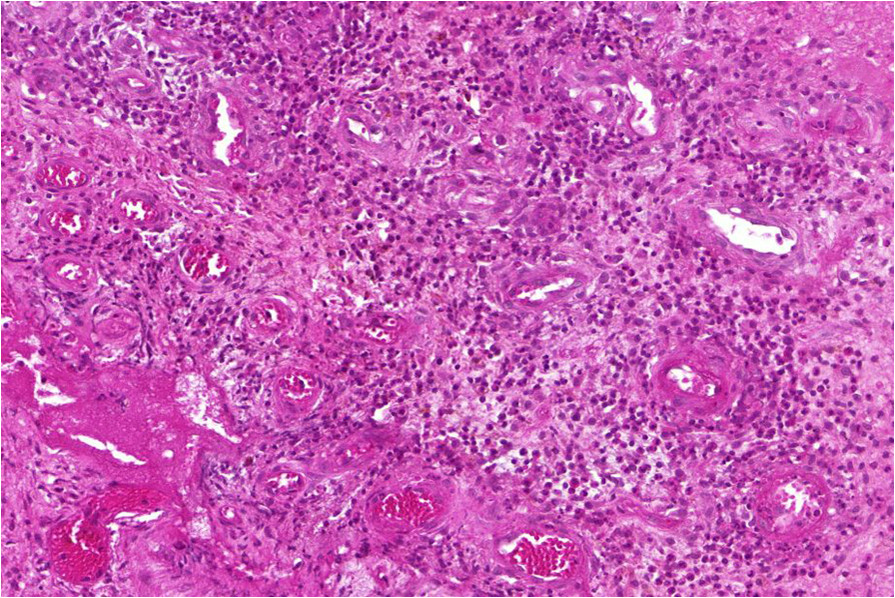
**Biopsy of oedematous forefoot lesion 2 weeks after commencing ART showing a dense inflammatory reaction with lymphoid granulomas consistent with a paradoxical reaction.**

**Figure 3:**
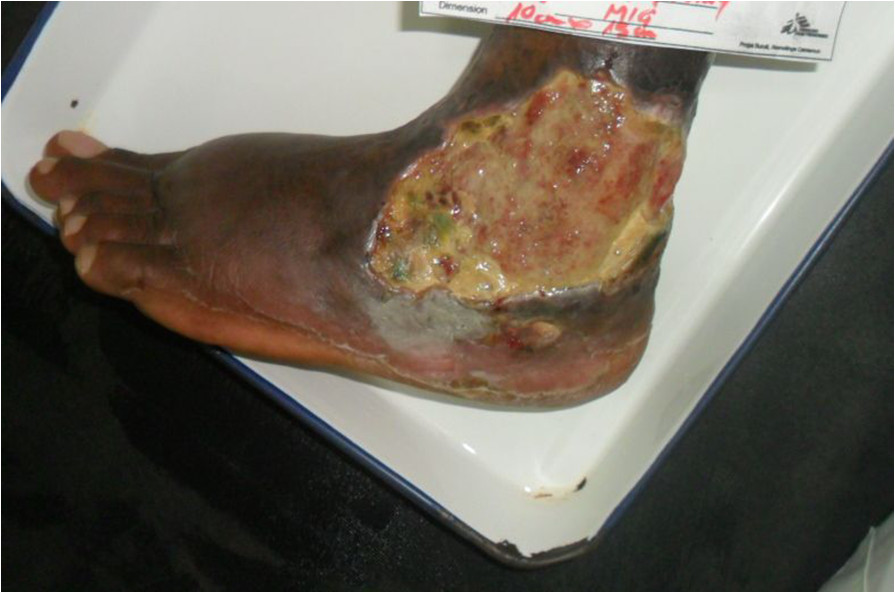
**BU ulcer on lateral aspect of the left ankle showing significant reduction in swelling of skin and subcutaneous tissue of the forefoot with preservation of tissue following 10 days of prednisolone treatment.**

**Figure 4:**
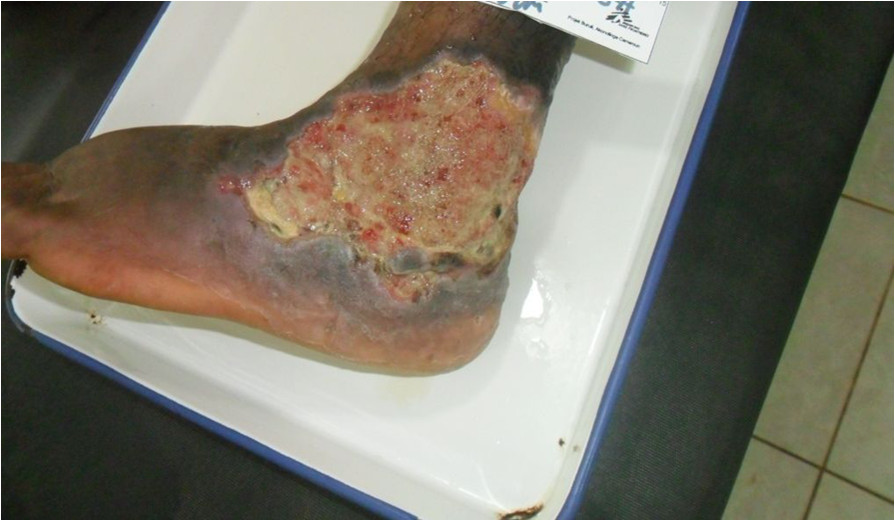
**BU ulcer on lateral aspect left ankle showing significant reduction in swelling of skin and subcutaneous tissue of the forefoot with preservation of tissue following 30 days of prednisolone treatment.**

Antiretroviral treatment (ART) was commenced on 19/1/13 with a regimen containing zidovudine, lamivudine and efavirenz. Co-trimoxazole 960 mg/day was also commenced at the same time. Antibiotic and ART adherence was directly supervised in the hospital.Two weeks after ART initiation the tissue around the ulcer became inflamed, with significant oedema extending across the dorsum of the foot to the toes with evidence of extending necrosis and ulceration from the wound margins. (Figure 1) A provisional clinical diagnosis of a severe paradoxical reaction was made and 2 tissue biopsy specimens were performed for histological and microbiological examination; one from the edge of the lesion and one from the centre of the oedematous dorsum of the foot. Whilst awaiting the results the patient was commenced on oral prednisolone (50 mg daily) with the aim to reduce the severity of the paradoxical reaction and prevent further ulceration of the tissue on the dorsum of the foot. Prednisolone was continued at 50 mg daily for 2 weeks then weaned to 30 mg daily for 1 week, then 20 mg daily for 2 weeks and then 10 mg daily for 2 weeks (total 7 weeks). Albendazole 400 mg daily for 3 days was given at prednisolone commencement to treat any possible strongyloides co-infection. ART was continued unchanged.Histopathological examination of the tissue biopsy revealed diffuse inflammation with lymphoid granulomas (Figure 2) and was negative on examination for acid fast bacilli (AFB) consistent with a paradoxical reaction. Mycobacterial cultures were not performed on the tissue. Following prednisolone treatment the tissue oedema rapidly settled and no further ulceration of the skin on the adjacent dorsum of the foot occurred (Figures 3 and 4). The prednisolone treatment was well tolerated.

**Figure 5:**
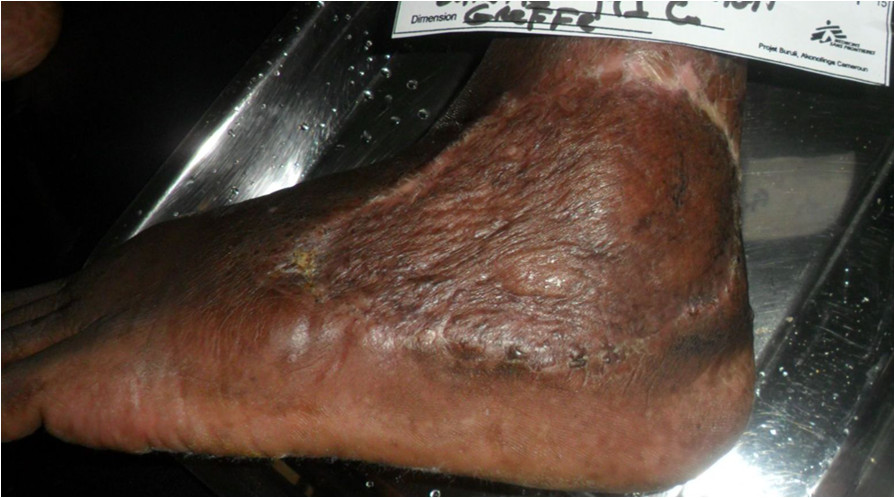
**Healed BU lesion 10 months after commencement of antibiotics and 2 months post split skin graft.**

**Figure 6:**
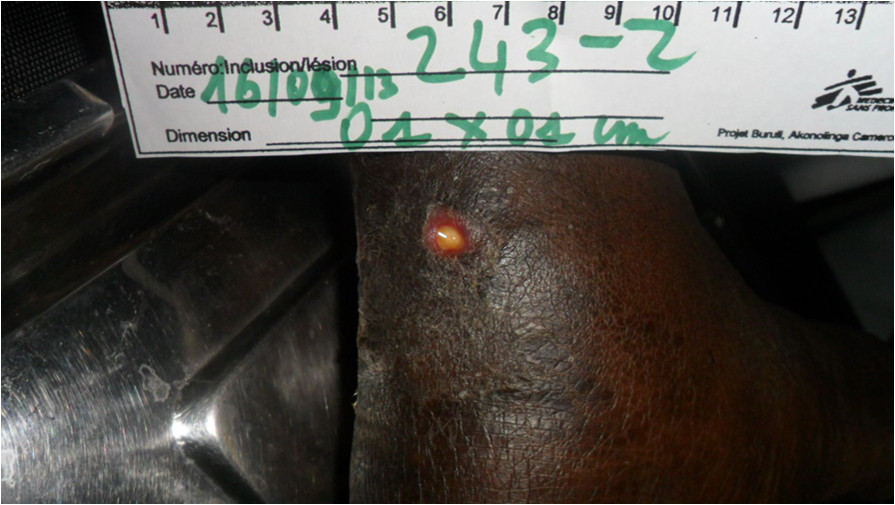
**Second paradoxical lesion developing in retromalleolar region of left ankle 8 months after commencing BU antibiotic treatment.**

The BU antibiotics were ceased on 3/3/13 after 53 days of treatment with no further adverse effects. The ankle lesion underwent surgical debridement to remove hypergranulation tissue on 13/3/13. The skin defect underwent further surgical debridement and was closed with a split skin graft on 4/7/2013. The graft healed well (Figure 5). The patient had excellent adherence to his ART which he tolerated well. After 6 months of ART the viral load was undetectable and after 8 months the CD4 count had increased to 416 cells/mm^3^.A further 1 cm ulcerated lesion on the retromalleolar region of the left ankle appeared 8 months after the start of BU antibiotic treatment. (Figure 6) This was diagnosed as a late paradoxical reaction clinically and was confirmed as such by a biopsy revealing significant inflammation with no AFB seen on histology, and mycobacterial cultures of a swab of the lesion were negative despite the PCR of the swab remaining positive. The lesion healed 12 weeks later with dressings as the only treatment.

The patient was discharged from hospital 11 months after starting antibiotics. Twelve months after completing antibiotic treatment all the lesions remain healed with no evidence of BU recurrence.

### Discussion of case

We present a case of a severe paradoxical reaction occurring in an HIV-infected patient receiving combined BU antibiotic treatment and ART. The severity of the reaction threatened the viability of tissue around the BU lesion, especially across the dorsum of the left foot. The diagnosis of a paradoxical reaction was initially based on a consistent clinical picture where the lesion and surrounding tissue deteriorated after 3 weeks of antibiotics [5, 6]. This was later supported by the histological appearances that showed evidence of an intense inflammatory reaction consistent with previous descriptions of paradoxical reactions [6, 9]. Paradoxical reactions, also known as immune reconstitution reactions, [9] are proposed to result from a reversal of the mycolactone toxin induced immune-inhibitory state via the antibiotic mediated killing of *M. ulcerans* organisms allowing an intense immunological reaction to develop against the persisting mycobacterial antigens [10, 11]. As has been reported in HIV-negative patients following the completion of antibiotics, [12, 13] a second paradoxical lesion developed on the retromalleolar region of the left ankle 6 months after antibiotics were ceased. Late reactions may account for up to 24% of paradoxical cases, [6] and this reaction was likely due to enhanced immune responses to a previously undetected disease focus.

It is not known whether paradoxical reactions are increased in HIV patients with BU, whether this is influenced by the baseline level of immune suppression or whether it is further potentiated when the generalised immune suppressed state is partially reversed by ART. However it is known that paradoxical reactions are common in HIV patients commencing ART with a variety of other microorganisms including tuberculosis (TB) [14], cryptococcus [15] and Mycobacterium avium complex [16], and that the rate increases with increasing immunosuppression at ART baseline [17]. Furthermore in TB/HIV co-infection, the incidence of TB associated immune reconstitution disease is increased in those who commence ART within 30 days of commencing TB treatment [14]. If these findings can be extrapolated to BU treatment, then in our case some factors existed that may have increased the paradoxical reaction risk: the baseline level of immune suppression was significant (CD4 220 cells/mm^3^) and ART was commenced early (2 weeks) after the start of BU treatment. Furthermore the good response to his ART with a robust increase in his CD4 cell count and reduction in viral load to undetectable levels may have contributed. A recent report from Ivory Coast also describes further multifocal BU lesions developing in a HIV positive patient with severe immunosuppression (baseline CD4 count of 51 cells/mm^3^) one month after starting both BU antibiotic treatment and ART [18]. Further studies on the incidence and risk factors for paradoxical reactions during treatment of patients co-infected with BU/HIV are needed to investigate these issues [8].

Oral prednisolone was used in this patient to settle a severe paradoxical reaction. We believe it prevented further skin and subcutaneous tissue loss on the dorsum of the foot, which commonly occurs during treatment of oedematous BU lesions and with severe paradoxical reactions [6]. Evidence from the mouse model suggests that the use of corticosteroids does not increase the risk of BU treatment failure, [19] and it has been used successfully in an Australian setting to minimise damage from severe paradoxical reactions [6, 7, 20]. Potential risks of prednisolone administration in an African setting, such as worsening TB or disseminated strongyloides infection, were minimised by active screening of the patient for TB and administration of albendazole at initiation of prednisolone treatment. The prednisolone was well tolerated and did not result in any adverse effects. The usual recommended dose is 0.5-1.0 mg/kg daily tapered over 4-8 weeks [21]. Of note, despite the use of prednisolone, the duration of BU antibiotic treatment was not prolonged and the antiretroviral regimen was not ceased or altered and this did not appear to adversely affect outcomes.

This case also illustrates a number of important issues relevant to the patient co-infected with BU and HIV. Firstly the patient presented with large, multiple and ulcerated lesions consistent with previous reports of more severe BU disease at presentation in those co-infected with HIV [2, 4]. Secondly, due to a hypersensitivity reaction to streptomycin, the patient was treated with the all oral antibiotic combination of rifampicin and clarithromycin with good success. Although oral combinations have been effective in observational studies of HIV negative patients with BU, [22, 23] this case provides preliminary evidence that they may also be effective in HIV co-infected patients. Thirdly, although effective and safe in this case, there are concerns regarding the interaction of clarithromycin and efavirenz that may lead to reduced effectiveness and increased toxicity of BU treatment [24]. This potential interaction requires further study.

## Conclusions

Severe paradoxical reactions can occur during the antibiotic treatment of BU/HIV co-infected patients. Prednisolone was effectively and safely used to settle the reaction and minimize the secondary tissue damage. Further study on the incidence, risk factors and management of paradoxical reactions in HIV/BU co-infected populations is needed.

## Consent

Written informed consent was obtained from the patient for publication of this Case report and any accompanying images. A copy of the written consent is available for review by the Editor of this journal.

## Authors’ original submitted files for images

Below are the links to the authors’ original submitted files for images. Authors’ original file for figure 1 Authors’ original file for figure 2 Authors’ original file for figure 3 Authors’ original file for figure 4 Authors’ original file for figure 5 Authors’ original file for figure 6
