# Supplementary figures and images for: Clinical features and management of a severe paradoxical reaction associated with combined treatment of Buruli ulcer and HIV co-infection
Source: BMC Infect Dis. 2014 Jul 30;14:423. doi: 10.1186/1471-2334-14-423 (PMC4122778; doi:10.1186/1471-2334-14-423)

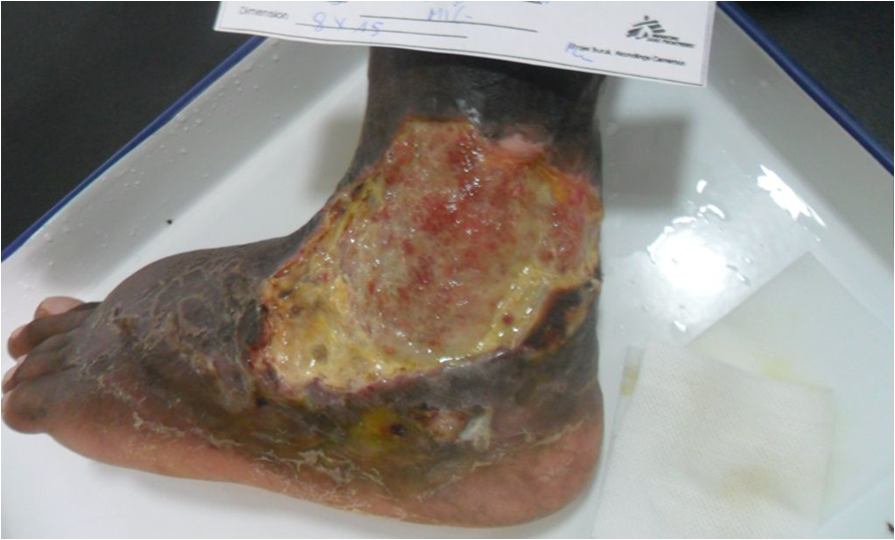

Supplement: Supplementary file 1 — Authors’ original file for figure 1 [file 12879_2014_3719_MOESM1_ESM.tif]

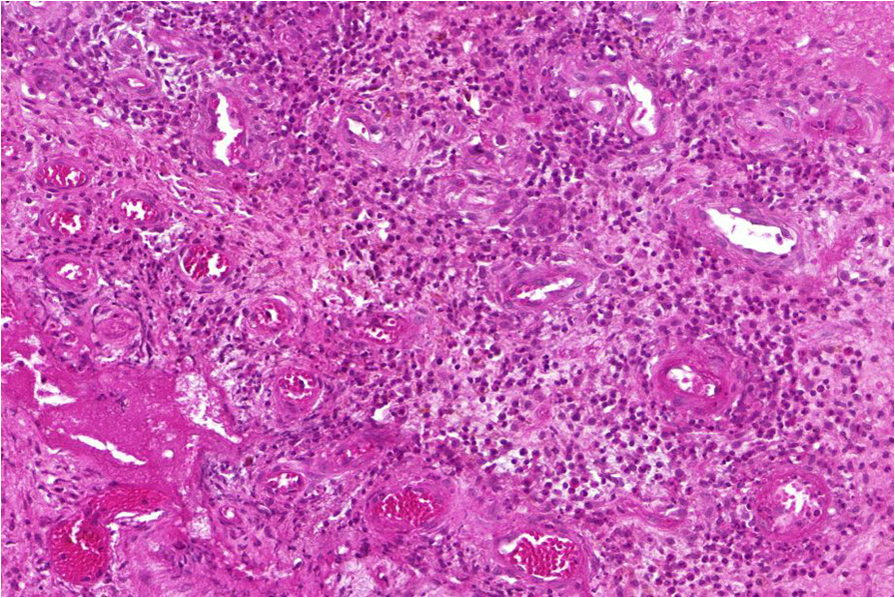

Supplement: Supplementary file 2 — Authors’ original file for figure 2 [file 12879_2014_3719_MOESM2_ESM.tif]

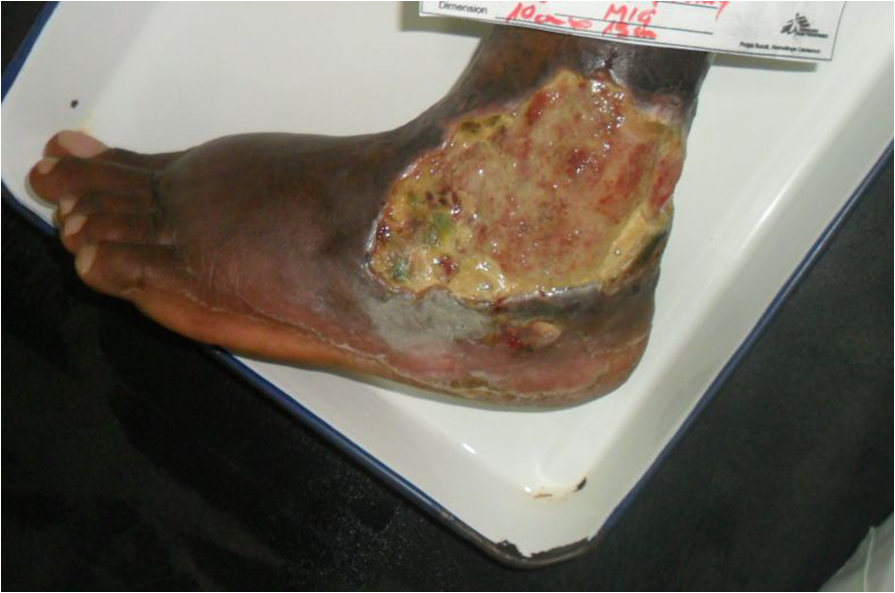

Supplement: Supplementary file 3 — Authors’ original file for figure 3 [file 12879_2014_3719_MOESM3_ESM.tif]

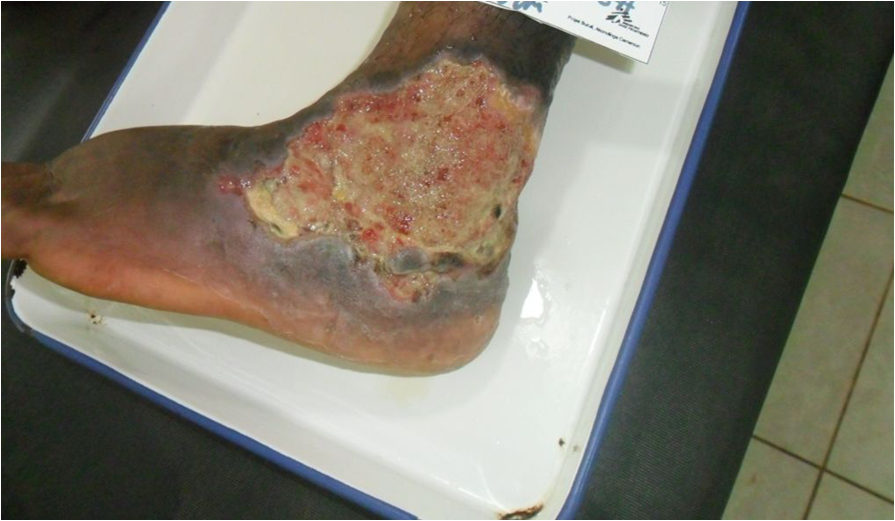

Supplement: Supplementary file 4 — Authors’ original file for figure 4 [file 12879_2014_3719_MOESM4_ESM.tif]

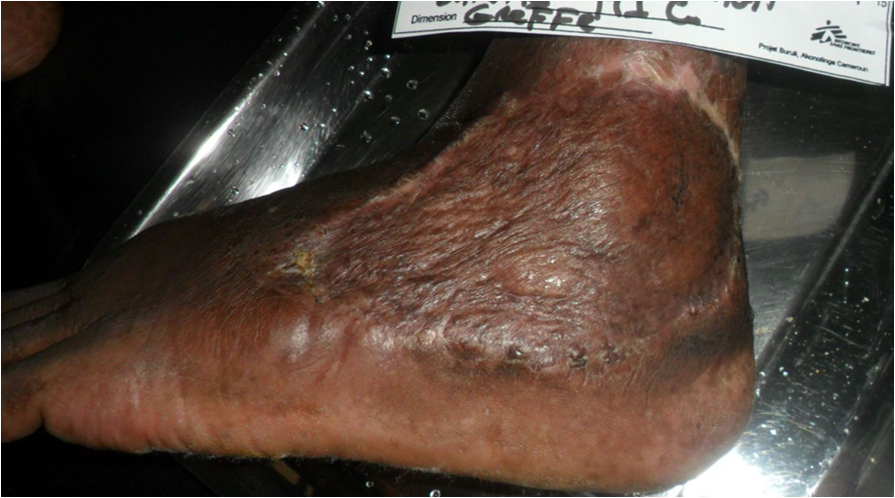

Supplement: Supplementary file 5 — Authors’ original file for figure 5 [file 12879_2014_3719_MOESM5_ESM.tif]

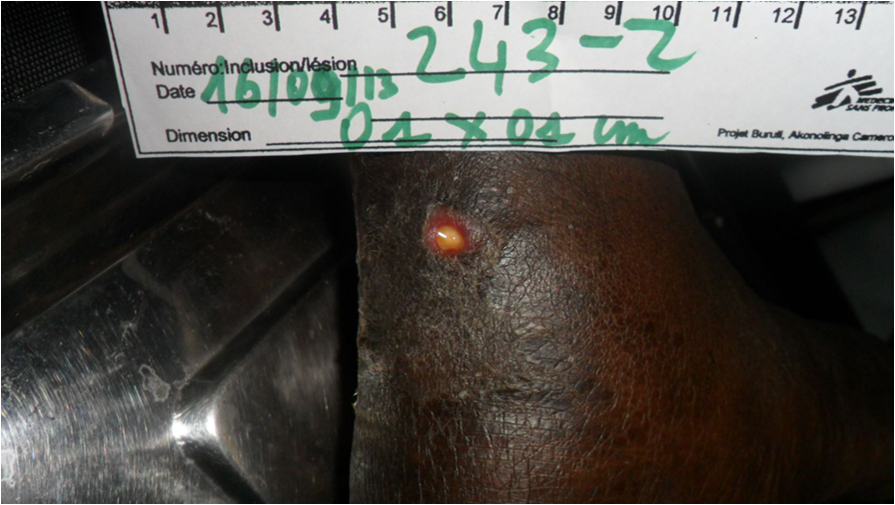

Supplement: Supplementary file 6 — Authors’ original file for figure 6 [file 12879_2014_3719_MOESM6_ESM.tif]
